# Eating disorders - knowledge, attitudes, management and clinical experience of Norwegian dentists

**DOI:** 10.1186/s12903-015-0114-7

**Published:** 2015-10-14

**Authors:** Ann-Katrin Johansson, Anders Johansson, Eva Nohlert, Claes Norring, Anne Nordrehaug Åstrøm, Åke Tegelberg

**Affiliations:** Department of Clinical Dentistry – Cariology, Faculty of Medicine and Dentistry, University of Bergen, Årstadveien 19, N-5009 Bergen, Norway; Department of Clinical Dentistry – Prosthodontics, Faculty of Medicine and Dentistry, University of Bergen, Bergen, Norway; Centre for Clinical Research, Västerås, Sweden; Uppsala University, Uppsala, Sweden; Stockholm Center for Eating Disorders, R&D Unit, Centre for Psychiatry Research, Karolinska Institutet/ Stockholm County Council, Stockholm, Sweden; Department of Clinical Dentistry – Community Dentistry, Faculty of Medicine and Dentistry, University of Bergen, Bergen, Norway; Postgraduate Dental Education Center, Örebro, Sweden; Faculty of Odontology, Malmö University, Malmö, Sweden

**Keywords:** Attitude, Dentists, Eating disorders, Knowledge, Questionnaire

## Abstract

**Background:**

The purpose of this study was to investigate knowledge, attitudes and clinical experience with regard to patients with eating disorders (ED) among Norwegian dentists.

**Methods:**

In 2010, a questionnaire was sent to all dentists in Norway (*N* = 4282) comprising 33 questions related to demographics of the participating dentists, their knowledge of ED (general and oral health aspects), clinical experience, attitudes and perceived management preferences.

**Results:**

The participation rate was 40 % (47 % women and 53 % men). Their knowledge about ED was often retrieved from common media sources and the greater part of the participants reported they had seen very few patients with ED during their professional career. Female dentists reported superior knowledge about ED compared to males, but the former experienced greater difficulties to inform about the condition. Referrals of the patient to other health facilities were significantly more common among female compared to male dentists. The majority of dentists (76 %) reported a need of more education related to ED management.

**Conclusions:**

The Norwegian dentists in this study reported limited clinical experience and insufficient knowledge regarding ED. There is therefore a need to increase both undergraduate and continuing education in this field, which can improve preventive and management measures that a dentist can provide for ED patients.

## Background

Eating disorders (ED) are psychosomatic disorders, and commonly also associated with impaired oral health. This may include, for example, dental caries and erosion in addition to impaired salivary function, parotid gland enlargement and temporomandibular disorders (TMD) [1–5]. A multidisciplinary approach, including the dental profession, is therefore necessary when providing comprehensive care to ED patients and its prevention; furthermore, the treatment of its oral manifestations can be important in the overall management and prognosis of ED patients [6–8].

Early detection of ED disease is considered to be of utmost importance for treatment outcome, as well as reducing the risk of somatic, psychological and/or oral complications [9]. In this regard, it is important to remember that ED is associated with feelings of guilt and shame in addition to denial of the illness. Indeed, patients with ED often hide the real origin of their problem or avoid contact with healthcare professionals [10, 11].

The dental team sees many patients regularly through its system of regular recalls. Thus it is important that they are able to identify the specific signs and symptoms of ED among their patients [12, 13]. Regretfully, studies have shown that dentists and dental hygienists often have an insufficient level of knowledge in this regard [14] and that it is common that they are reluctant to inform the patient/parents even if they suspect that their patients suffer from ED [15, 16].

Guidelines for management of ED patients were published in Norway (2000) and Sweden (2005). These recommendations included general oral health considerations but no specific guidelines regarding dental management of patients with ED have to date been established [17, 18]. A previous study performed among Swedish dentists found that the knowledge and clinical experience as regard patients with ED were poor. The conclusion was drawn that education in this area needs to be improved, which would have the potential to encourage dentists to become more involved in both secondary and tertiary prevention and management of ED [19].

The neighboring country, Norway, which has a similar demography when it comes to education and the practice of dentistry, has no data available as to the awareness of ED among dental health care providers. The aim of this study was therefore to investigate the level of knowledge, attitudes, management and clinical experience of Norwegian dentists in relation to ED.

## Methods

The methodology applied in this study is to a great extent a duplication of a previous study performed in Swedish dentists [19].

### Subjects

A questionnaire was sent by ordinary mail to all dentists in Norway registered in the membership list of the Norwegian Dental Association (*n* = 4282).

### Questionnaire

The questionnaire comprised 33 questions or statements based on a previous study [19]. Most of them had a multiple choice format but in six the subjects were requested to give an open answer. The questions comprised socio-demographic and personal data, including year of birth, gender, and graduation year from dental school. Other domains encompassed knowledge of eating disorders from general and oral health perspectives, attitudes towards the disease as well as the dentists’ experience of ED and perceived management options. The questionnaire was delivered by ordinary post during spring 2010. Non-responders received one reminder.

### Piloting the questionnaire

A convenience sample of ten independent dentists (general practitioners and specialists) was asked to respond and comment on the questionnaire and the final questionnaire was adjusted accordingly.

### Ethics

The project was submitted to the Regional Committee for Medical and Health Research Ethics (REC West, Norway) but the Committee judged that the project did not need formal ethical approval (REK Vest no. 2010/905–2). The project was registered and approved by the Norwegian Data Protection Office (NSD no. 24040). Informed consent from the participants was not required according to the response from the Ethical committee (REK Vest no. 2010/905–2) and the Norwegian Data Protection Office NSD (NSD no. 24040).

### Statistical analyses

Descriptive statistics were carried out. The Mann-Whitney *U*-test was applied when comparing continuous, ordinal and numerical/skewed variables between 2 groups (e.g. gender and length of professional experience). Pearson Chi-Square tests were applied when analyzing proportions for categorical variables. Wilcoxon Signed Rank Test was used for paired observations. All statistical analyses were calculated using IBM SPSS statistics, version 21. p-values less than 0.05 were considered statistically significant.

## Results

### Response rate and demographics

The overall response rate was 40 % (1726/4282). The majority of responding dentists were aged 50–66 years (42 %) and in decreasing order, 30–39 years (26 %), 40–49 years (21 %), >66 years (6 %) and <30 years (5 %). Gender distribution was 47 % women and 53 % men (not in tables). The distributions of the respondents as regard professional affiliation and workplace, number of years spent working ≥ 5 years as a dentist are shown in Table 1. There were significantly more males than females working in private practice (41 % females vs. 59 % males) while in Public Dental Health Service (PDHS) there were more females (66 % females vs. 34 % males) (*p* < 0.001, Table 1). There was no statistically significant difference as regard the length of professional experience (>5 years) of general PHDS practitioners (87 %), private practitioners (93 %) and senior consultants working (98 %) (Table 1). The proportions of all responders as regards affiliation to private sector or PDHS was 72 and 28 % respectively, while the corresponding figure for non-responders were 68 and 32 % (*p* < 0.01) (not in tables). No other variables between responders vs. non-responders could be analyzed due to the restrictions set by the Norwegian data protection authority and ethical committee.

**Table 1:** Professional affiliation/workplace, gender distribution and professional experience among the dentists

	Total		Women		Professional experience ≥ 5 years	
	N	%	N	%	N	%
General PDHS^α^ practitioner	381	25	252	66	331	87
General private practitioner	936	61	388	41	870	93
Senior consultants^β^	205	15	77	38	201	98
Total^γ^	1522	100	717	47		

^α^PDHS = Public Dental Health Service

^β^Includes consultants and dentists attending specialist training, working in both PDHS and private practice

^γ^The number of responders (1522) differ with respect to the total number of participants (*n* = 1726) due to internal missing data (204 did not respond to the question)

### Knowledge

The self-rated general knowledge about ED was significantly better among females than males (*p* < 0.001; Table 2). There was no significant difference between different professional affiliations (general practitioners or senior consultants) with respect to perceived general knowledge about ED (not in tables). Dentists who had worked <5 years perceived their knowledge as significantly better than those who had worked for 5 years or more (*p* < 0.05) (not in tables).

**Table 2:** Self-rated general knowledge about eating disorders among dentists by gender

	Females			Males	
Self-rated general knowledge	N	%	p	N	%
Very Good	75	10	<0.001	31	4
Good	264	35		232	28
Relatively good	305	41		358	43
Poor	103	14		209	25
Very poor	2	0.3		9	1
Total^a^	749			839	

p denotes difference between females and males (Mann-Whitney *U*-test)

^a^Total responders 1588, 138 non-responders

Sources of the dentists’ acquired knowledge regarding general and oral health in patients with eating disorders are presented in Table 3. The most frequently given source was media. Females reported significantly more often than males to have acquired knowledge from media (females 71 % vs. males 64 %; *p* < 0.01) dental school (females 58 % vs. males 45 %; *p* < 0.05), through courses related to ED (females 21 % vs. males 17 %; *p* < 0.05) and from other sources (females 21 % vs. males 17 %; *p* < 0.05) (not in tables).

**Table 3:** Sources of acquired knowledge regarding general and oral health in patients with eating disorders (more than one response alternative was allowed)

	Knowledge in general health			Knowledge of oral health	
Source of acquired knowledge	N	%	*p*	N	%
Media	1153	68	< 0.001	576	34
Own experience	661	38	< 0.001	974	58
Dental school	673	39	< 0.001	861	51
Self-studies	460	27	NS	454	27
Courses	282	16	< 0.001	317	19
Other sources	316	18	< 0.01	269	16

*p* denotes difference between general and dental knowledge (Wilcoxon Signed Rank Test)

As regard knowledge of ED and oral health implications, dentists with shorter experience (<5 years) had gained their knowledge significantly more through their undergraduate training (92 % vs. 47 %; *p* < 0.001) (not in tables). Those with longer professional experience had obtained more of their knowledge through media (35 % vs. 18 %; *p* < 0.001), own experience (59 % vs. 45 %; *p* < 0.01), self-studies (28 % vs. 11 %; p < 0.001), continuing dental education (20 % vs. 5 %; *p* < 0.001) and other sources (17 % vs. 7 %; *p* < 0.01) (not in tables).

The most common perceived causes for developing ED were psychological (92 %) and socio-cultural factors (75 %), while heredity and biological factors as a cause of ED was only reported by 9 % and 8 % of the respondents, respectively (not in tables). Females perceived significantly more frequently than males that biological (females 19 % vs. males 6 %; *p* < 0.05) and socio-cultural (females 78 % vs. males 73 %; *p* < 0.05) factors played a role in developing ED. Dentists with shorter working experience (<5 years) believed more frequently than those with longer experience that psychological (97 % vs. 92 %; *p* < 0.05) and socio-cultural (85 % vs. 74 %; *p* < 0.01) factors were important for developing ED (not in tables). There were no differences in these aspects regarding professional affiliation (general practitioners or senior consultants).

### Attitudes and management

Four percent of respondents felt that the dental care system provided sufficient means for managing ED patients, while 45 % considered that they did not get adequate help in the matter and 51 % did not know. Eighty-five percent believed that ED patients had more dental problems than other patients. The majority of respondents (75 %) thought that dental treatment would be of great/very great benefit in the overall medical management of ED patients, but 76 % reported that in order to provide such care they would need more training (not in tables). Females and general practitioners believed more frequently than males (78 % vs. 72 %; *p* < 0.01) and senior consultants (75 % vs. 71 %; *p* < 0.05) that dental treatment would benefit the general management of ED patients (not in tables). In addition, general practitioners considered significantly more frequently than senior consultants that they needed more training in the management of ED (78 % vs. 71 %; *p* < 0.05). The level of difficulties expressed by dentists when having to inform a patient (or a parent of a patient) with suspected ED are shown in Table 4. Females found it more difficult to do so when an actual situation occurred than males (*p* < 0.01, Table 4) and dentists with shorter professional experience found it more difficult than those with longer experience (*p* < 0.05; not in tables).

**Table 4:** Level of difficulties expressed in informing patients with suspected eating disorder, and/or their parents, about the situation

	Females			Males	
	*N* = 750			*N* = 851	
Level of difficulties	N	%	*p*	N	%
No, never difficult	30	4	< 0.01	72	8
Yes, sometimes difficult	261	35		269	32
Yes, often difficult	181	24		173	20
Yes, always difficult	168	22		158	19
Never been in the situation	110	15		179	21
Total	750			851	

*p* denotes difference between females and males in the first 4 categories. (Mann-Whitney *U*-test)

The responses to possible management options when a dentist suspects that a patient has ED are shown in Table 5. Female dentists informed patients/relatives significantly more frequently and made recommendations more often to seek other medical care than males (*p* < 0.01). General practitioners treated ED patients as an ordinary patient until sure about the diagnosis more frequently than senior consultants (51 % vs. 34 %; *p* < 0.001) while the senior consultants referred the patients to other medical facility more frequently compared to general practitioners (21 % vs. 15 %; *p* < 0.05) (not in tables). Dentists with longer working experience (≥5 years) treated ED as ordinary patients significantly more frequently than those with shorter experience (24 % vs. 16 %; *p* < 0.05) (not in tables). Eighty percent of respondents considered that general dentists should manage these patients, followed, in decreasing order, by those who felt that dental healthcare professionals with special training for ED patients (50 %), dental hygienists (39 %), senior consultants (12 %) and hospital dentists (4 %) should do so (more than one response alternative was allowed) (not in tables). Females preferred more than males that ED patients should be managed by dental healthcare professionals with special training for ED patients (53 % vs. 46 %; *p* < 0.01) (not in tables).

**Table 5:** Suggested management options for a patient with a suspected eating disorder (ED)

	Females			Males	
	*N* = 750			*N* = 851	
Suggested management options	N	%	p	N	%
Treat as an ordinary patient	157	21		208	25
Treat as an ordinary patient until sure about ED	375	50		397	47
Inform patient or relatives (in children)	406	54	*P* < 0.01	398	47
Recommend the patient to seek other medical care	214	28	*P* < 0.01	187	22
Refer to:					
Senior dental consultants	30	4		30	3
Medical care^a^	108	14		133	15
Psychologist	39	5		42	5

More than one response was allowed. p denotes difference between females and males (Pearson Chi-Square)

^a^General medical practitioner

### Experience of ED patients

Over the preceding year, 56 % of respondents had been informed by a patient or the patient’s parent that he or she had an ED. For the whole group, the average number was 2 patients (range 1–30). Ninety-one percent confirmed that they had treated ED patients in their dental clinics during the preceding year, with a majority (*n* = 913 dentists) having treated 5 or fewer such patients (mean = 8 patients, range 1–90); the average age of these patients was estimated by the dentist to be 24 years and 88 % to be female. Forty-six dentists (3 %) had received referrals/consultations of between 1 and 30 ED patients during the preceding year and 155 dentists (9 %) had referred ED patients (data in the above paragraph not in tables).

Forty-six percent of the group had not suspected any diagnosis of ED during the preceding year, a finding that was more common among males (52 % vs. 39 %; *p* < 0.001), senior consultants (57 % vs. 44 %; *p* < 0.001) and in those with longer working experience (>5 years, 47 % vs. 28 %; *p* <0.001). Among the 54 % of dentists, who had suspected ED in a patient, there were 1887 instances where the patient/parent was not informed about the suspicion by the dentist; 953 instances where the dentist told the patient/parent and had the diagnosis confirmed, and 699 instances where the dentist told the patient/parent but not had the diagnosis confirmed. On this aspect, no differences were seen between genders, professional affiliation (general practitioners or senior consultants) or professional experience (not in tables). Dentists’ experiences of ED patients are summarized in Table 6.

**Table 6:** Percentage distribution of responses to questions relating to dentists’ experience of eating disorder (ED) patients

Question		Never/sometimes	Often	Very often/always	p
Supposed reaction from ED patients when informed that her/his oral status could be indicative of ED					
	Get insulted	68	23	9	
	Denies ED	44	30	26	<0.01^α^
	Seeks other dentist	88	9	4	<0.05^α^
	Admits ED	61	28	11	<0.01^β^
	Feels good that someone has understood	45	40	16	
	Seeks additional help for ED	65	24	11	
	Gets a chance for improved oral health	22	46	32	
	Doesn’t mean anything/no reaction	84	13	4	<0.05^β^
The dentists’ experience of ED patients as dental patients					
	Difficult to get in contact with	76	19	5	
	Difficult to get along with	81	15	5	
	Difficult to treat	73	20	7	
	Wants to talk about their ED	81	14	5	
	Listen to information	33	43	23	
Frequency of estimated problems experienced by ED patients					
	Aesthetics	19	41	40	<0.001^α^
	TMD	57	29	14	<0.001^α^
	Dentine hypersensitivity	15	42	43	<0.001^α^
	Dental fear	46	32	21	
	Fear for detection of the ED from the dental team	56	31	13	<0.01^α^
	Fear for high dental costs	29	36	34	
	Fear to speak about their ED with the dentist	46	37	17	<0.05^α^
	Worry for what may happen during dental treatment	50	35	15	<0.05^α^

p denotes difference between females and males (Mann-Whitney *U*-test)

^α^Significantly more female dentists responded often/very often/always than males

^β^Significantly more male dentists responded often/very often/always than females

About one-third of the respondents (35 %) believed that they could detect an anorectic person in a setting outside the dental office, while the corresponding figure for a bulimic person was 4 % (not in tables). Two out of three dentists in the study personally knew someone with ED (70 %), and most commonly it was a friend. Fifty-one responders (3 %), 47 females (6 %) and 4 males (0.5 %) reported having suffered from an ED themselves (not in tables).

## Discussion

One of the main findings of this study was that the responding dentists had a limited clinical experience in the management of ED. As ED is not an infrequent condition, it is in general of importance for dentists to evaluate and reach a better level of knowledge in this field through education and training.

In a recent Finnish study, lifetime prevalence and current ED diagnosis in young females and males (aged 20–35 years) were 6 and 2 %, respectively, and it was concluded that ED was the fourth largest group of mental disorders among young women and that the dentists’ exposure to ED patients was higher than reported [20]. Since it is clear that many patients with a diagnosis of ED do not inform the dentist (or anyone else) about their condition, it is likely that dentists relatively frequently treat dental patients without being aware that they have an ED.

The majority of dentists in our study rated their knowledge regarding ED to be relatively good to very good. However, the majority had only treated an average of 5 patients with ED or less during their professional lives. This indicates a generally unsatisfactory and limited clinical experience of patients with ED, in similarity to previous studies [14, 19]. The female dentists rated their level of knowledge significantly better than the males and in general the levels were higher than in a similarly-designed Swedish study [19]. The finding of superior self-rated general knowledge about ED among female compared to male dentists has been reported previously [16]. Female dentists also reported greater difficulty than male dentists in informing the patient/relatives about their ED suspicions. It is well known that eating disorders are significantly overrepresented among women. What is not as well known is that cognitions and attitudes associated with eating disorders, but without the presence of a diagnosable eating disorder, are very common in women [21]. During adolescence and early adulthood, many women undergo a period characterized by such thoughts and feelings. Women consequently know more about, but also to a greater extent identify themselves with, eating disorder problems. This could very well explain why female dentists find it more difficult to inform patients about a possible eating disorder [21].

The knowledge of the dentists in the present study was largely obtained from sources like media or own experience. Dentists with shorter working experience reported significantly better knowledge compared to those with longer experience. The former had most likely had teaching of ED included in their undergraduate curriculum and thereby gained more knowledge from dental school compared to the latter. This finding may reflect positively on modern dental education, but it has to be remembered that Bulimia Nervosa, as one of the most common ED diagnoses, was first established in 1979 [22] and consequently could not have been included in older dental curricula.

Only about half of the dentists felt that the patient/relative should be informed in a case of suspected ED. A reluctance by dentists and dental hygienists to convey such information has been reported earlier [15, 16]. This may result in the chance for early detection of the disease being lost, which is deemed to be very important for a successful management of ED [9]. In this regard it is important to remember that, among those dentists who informed the patient/guardian about their suspicion of ED, about half of the patients confirmed the suspicion when being asked. Among the other half of patients who did not confirm the ED diagnosis, this information provided by the dentist may well be taken as a warning sign from a *de facto* ED patient and hopefully lead her/him to seek treatment later in another setting.

Of the total number of responders, 6 % of female dentists and 0.5 % of males reported that they had suffered from ED themselves, which roughly corresponds to the estimated prevalence and gender distribution within the general population (20).

One Swedish survey concluded that a more structured cooperation between the dental team and other actors involved in the management of ED patients should be implemented [23]. In the present study, relatively few dentists recommended the patient to seek other medical care or referred them to other health care facilities, similar to that found in the Swedish study [19]. In Norway, the recommendation for ED patients is to be referred mainly to the general medical practitioner or a psychologist. As regard dental management of ED patients, only 4 % of the respondents believed that the dental care system in Norway provided adequate help. Nevertheless, it seems to be of utmost importance that dentists in Norway are informed about the available alternatives for management of ED patients.

The majority of dentists were aware of they had treated ED patients during the preceding year, although the majority of them had only encountered a few patients in their professional lives. In addition, most of the dentists believed that ED patients had more oral complications than an ordinary patient. Therefore, it was not an unexpected finding that the majority reported that they needed more training in the dental management of ED patients. This supports conclusions from previous studies and suggestions to implement more training in the management of ED patients in undergraduate, postgraduate as well as in continuing dental education [19, 23, 24].

The ability to generalize from the results of a questionnaire depends on sufficient number of responders. A response rate of at least 60 % is set as a minimum requirement for publication by some scientific journals [25]. However, there is a steady decline in response rates in published surveys of health care providers in the USA, and during 2005–2008 only about 35 % met the 60 % criteria and none in 2009 [26]. This was also true for postal surveys of healthcare professionals covering 1996 to 2005, where the response rate (350 studies, average response rate 58 %) was significantly lower than during the previous 10-year period. It was even lower in studies with more than 1000 participants. The conclusion drawn in 2005 was that response rates to postal surveys of healthcare professionals were low and probably declining, which may lead to unknown levels of bias [27]. In line with the forgoing, it was not surprising that we only reached an overall response rate of 40 % in this study that was performed in 2010 and having a high number of selected participants (*N* = 4282). The reasons for low response rate to questionnaires and strategies to overcome such problems have been thoroughly elaborated on in a systemic review where 110 different approaches were assessed with the conclusion drawn that there are many ways to increase the response rate in questionnaire surveys [28].

If the number of responders is inferior to the number of non-responders, it will be a weakness in the interpretation of the conclusions. The low response rate in the present study makes it difficult to draw valid conclusions for the total population of Norwegian dentists. On the other hand, the questionnaire was sent to all Norwegian dentists and the responders represent one specific professional occupation. The questionnaire included specific work related questions and statements, which allow meaningful correlations and conclusions to be drawn. Because of restrictions set by the Norwegian data protection authority and ethical committee, the only non-response analysis that could be performed was related to work affiliation.

## Conclusions

The participating Norwegian dentists in this study reported limited clinical experience and insufficient knowledge regarding ED and its treatment in analogy with a similarly-designed study among Swedish dentists [19]. It is therefore a need to increase both undergraduate and continuing education in this field, which can improve preventive and management measures that a dentist can provide for ED patients.

## Abbreviations

ED: Eating disorders
TMD: Temporomandibular disorders
PDHS: Public Dental Health Service
